# Impact of provider feedback on quality improvement in rehabilitation services: an interrupted time series analysis

**DOI:** 10.3389/fresc.2025.1564346

**Published:** 2025-03-06

**Authors:** Anne-Lene Sand-Svartrud, Hanne Dagfinrud, Johanne Fossen, Heidi Framstad, Eirik Lind Irgens, Hild Kristin Morvik, Joseph Sexton, Rikke Helene Moe, Ingvild Kjeken

**Affiliations:** ^1^ Health Services Research and Innovation Unit, Centre for Treatment of Rheumatic and Musculoskeletal Diseases (REMEDY), Diakonhjemmet Hospital, Oslo, Norway; ^2^ HLF Rehabilitation, Oslo, Norway; ^3^ Røysumtunet Rehabilitation Centre, Jaren, Norway; ^4^ Helsepartner Rehabilitation Centre, Alta, Norway; ^5^ Åstveit Health Centre, Åsane I Bergen, Norway; ^6^ Division of Rheumatology and Research, Centre for Treatment of Rheumatic and Musculoskeletal Diseases (REMEDY), Diakonhjemmet Hospital, Oslo, Norway

**Keywords:** quality of health care, rehabilitation, quality indicators, feedback, clinical audit

## Abstract

**Introduction:**

Quality improvement in rehabilitation is needed due to unwarranted variations and suboptimal service coordination. Audit and feedback strategies are commonly used to improve healthcare quality, but evidence of their effectiveness in rehabilitation settings is limited.

**Objective:**

To evaluate the impact of an audit and feedback strategy on rehabilitation quality, as measured by a set of quality indicators (QIs) specifically designed for rehabilitation.

**Methods:**

Interrupted time series analysis was conducted across 16 Norwegian institutions delivering specialized rehabilitation for long-term diseases. Patient-reported rehabilitation quality data was collected continuously before and after a provider feedback intervention, while provider-reported quality was measured once before and after the intervention. We compared 11 pre- and 9 post-intervention observations, each spanning 3 weeks, over a 15-months study period.

**Results:**

The analyses included 2,415 patients, with 1,444 (59.8%) pre-intervention and 971 (40.2%) post-intervention. Mixed model analyses revealed that the mean differences in patient-reported QIs between the pre- and post-intervention phase were small and statistically non-significant. The expected impact model, including a gradually higher quality after the feedback to institution managers and clinical team members, was not confirmed. We observed variations in service quality among institutions, also post-intervention. The lowest pass rates were observed for indicators addressing the follow-up, involvement of external services and next of kin.

**Conclusions:**

In this multicentre study, the audit and feedback intervention did not lead to improvements in the quality of rehabilitation services, as measured by changes in QI pass rates covering health service structures, processes and patient outcomes.

**Clinical Trial Registration:**

ClinicalTrials.gov [NCT03764982].

## Introduction

Rehabilitation services may result in individual and societal benefits for the increasing proportion of people living with disabilities due to long-term diseases (1). The need for such services is substantial and growing (2), yet current evidence on the effectiveness of multidisciplinary, non-pharmacological rehabilitation in improving patient health outcomes remains inconclusive (3–6). Given that some of these outcome variations may relate to differences in professional rehabilitation practice, actions are needed to ensure delivery of high-quality rehabilitation services to all patients (1, 2, 7).

In Norway, secondary healthcare rehabilitation is delivered in hospitals or private rehabilitation institutions, with subsequent follow-up in primary care (8). Public evaluation reports conclude that the quality of rehabilitation services is characterized by unwarranted variations among institutions and services, in terms of fragmented interventions that are insufficiently coordinated across professions, services and healthcare levels, and with a suboptimal level of patient involvement (9, 10). In contrast, high-quality rehabilitation practice should reflect a multi-step process tailored to each individual patient, with coordinated interventions involving different professions and services, often provided along a continuum of care from hospitals or institutions in secondary healthcare to rehabilitation in local municipalities (11). It is essential to find ways to evaluate and improve each step, as well as the process as a whole.

Audit and feedback strategies are widely used to evaluate and improve the quality of healthcare services (12–15). These strategies include systematic assessments of various aspects of healthcare delivery, such as clinical performance in patient treatment, existing procedures, administrative structures, and patient outcomes (12–15). Systematic feedback to health professionals and managers regarding their adherence to established standards, can enable them to pinpoint areas for refinement, address shortcomings, and optimize their services (12–15). The overarching objective of audit and feedback strategies is to motivate clinicians and institution managers to sustain practices aligned with criteria for high-quality care, and, if necessary, to prompt them to identify and carry out actions for improvement in areas of suboptimal care delivery (15).

Quality indicators (QIs) may have a crucial role in audit and feedback strategies, as indicators reflect standards of care based on the best available, scientific evidence, guidelines, recommendations, and expert opinions (16, 17). Utilization of QIs allows the measurement of a set of identifiable events that are expected to occur during delivery of high-quality health services (16–19). When included in audit and feedback strategies, QIs can work as benchmarks for evaluating different dimensions of healthcare quality, including structures, processes of care, and patient outcomes. In a specific QI set developed in 2019 for use in rehabilitation, the structure indicators measure the settings in which the rehabilitation occurs, in terms of available, written procedures defining care intended to be provided (20). The process indicators measure what is actually done in providing and receiving care, and the outcome indicators measure the patient perspective on meaningful improvements in goal-attainment, function, and well-being (20).

Audit and feedback strategies are used in various ways from local initiatives to mandatory assessments initiated by national or international health authorities (15, 21). However, there is a paucity of studies investigating the effectiveness of audit and feedback on quality improvement in rehabilitation services, and previous work has mostly focused on rehabilitation quality in intensive care units (22).

More knowledge is needed on methods for providing feedback on provider performance in rehabilitation services for people with long-term diseases, as well as the impact of audit and feedback strategies in this context. Therefore, the aim of this study was to assess the impact of an audit and feedback strategy targeting healthcare providers on the quality of rehabilitation services, as measured by a QI set designed for use within the field of rehabilitation (20).

## Materials and methods

### Clinical setting and design

This study was part of the longitudinal RehabNytte Cohort (23), from which we included 16 out of 17 Norwegian institutions delivering multidisciplinary rehabilitation services to adults referred to rehabilitation in secondary healthcare due to various long-term diseases. Additional information about the relationship between the current study and the RehabNytte Cohort is given in Textbox 1.

Textbox 1 **The relationship between the current study and the RehabNytte Cohort.**

The RehabNytte (RehabBenefit) Cohort is a Norwegian longitudinal cohort study developed to monitor patient engagement, rehabilitation quality, and patients’ progress and benefit of rehabilitation services in secondary healthcare with regards to work ability, health, functioning, and well-being.

Participants: Eligible participants were adults (various diagnoses) referred to multidisciplinary rehabilitation in secondary healthcare and admitted to one of 17 institutions being part of the VIRKE Rehabilitation Research and Development Network. Data was collected at admission (T1), discharge (T2), and after 3 (T3), 6 (T4), and 12 (T5) months, through a digital data collection system with level 4 data security. The inclusion period was from January 2019 to March 2020. Data collection was completed in June 2021.

Interventions: Rehabilitation programs focusing on managing symptoms and consequences of long-term diseases were tailored for various patient groups at each institution. A team of at least four health professionals, typically physiotherapists, occupational therapists, nurses, and physicians, delivered the interventions. In some cases, a social worker, psychologist, sports educator, and/or nutritionist/dietitian was involved. The programs included groups and individual sessions, combined with self-training, primarily as inpatient rehabilitation for 2–3 weeks. Key topics covered self-management, physical training, and daily activities. Patient education and counselling addressed activity pacing, planning and adaptations of activities, coping strategies for pain/fatigue/sleep/stress, lifestyle changes (such as physical exercise, weight control, and smoking cessation), disease information, and medication. Other topics were adaptation of work or study activities, family and social relationships, and entitlements to social services.

Study design in the current study: The interrupted time series design in this study was planned as an independent study within the RehabNytte Project, aiming to assess the impact of an audit and feedback strategy, targeting healthcare providers, on the quality of rehabilitation services. Patients were recruited from all RehabNytte institutions that utilized the quality indicator set (16 out of 17 institutions).

In this paper, we refer to audit as the process of using the QI set for rehabilitation (20) as normative criteria for review of clinical practice and as a measure of professional performance, such that pass rates reflect the percentage of indicators that are successfully met (15, 24). We refer to feedback as a subsequent summary of the audit results that is reported back to clinicians and their managers, in terms of both separate data for each participating institution and aggregated data for comparison to other institutions included in the RehabNytte study (15, 24).

Using the QI set for multidisciplinary rehabilitation (20) as audit criteria, we evaluated whether the performance of the rehabilitation institutions and providers met the recommended standard of high level patient involvement throughout the rehabilitation process. It encompassed patient involvement in initial assessments, goal setting, and development of rehabilitation plans, as well as involvement of next of kin and external services. Additionally, it included adjustments of goals or interventions, and evaluation of progress conducted through team meetings and standardized assessment instruments (20). These topics were evaluated by one questionnaire completed by providers (addressing 19 structure indicators) and one completed by patients (addressing 11 process indicators and three outcome indicators) (Table 1). As the content of several of the structure and process indicators are matched, the QI set allows for evaluating and comparing quality from both the provider and the patient perspective (20) (Table 1). The set reflects the general rehabilitation process, making it applicable to programs focusing either physical rehabilitation, management training, psychosocial issues, lifestyle modifications, and patient education, determined by a patient-specific, goal-directed process involving several healthcare professionals over time.

**Table 1:** The quality indicator set for use in rehabilitation (20).

Main themes	Structure indicators (provider-reported): question (yes/no)	Process indicators (patient-reported): question (yes/no)
Patient participation in goal setting and rehabilitation process	S01. P shall participate in setting rehab goals.	P04. Were you actively involved in setting specific goals for the rehab period?
	S02. P shall participate in planning his/her rehab process.	
	S03. A template is used to prepare an individual rehab plan for P.	P03. Was a written plan developed for the rehab period (comprising your rehab goals, what you should practice, etc.)?
		P05. Were you actively involved in preparing a specific written plan for the rehab period (mentioned in q. 3)?
	S04. P shall participate in evaluating his/her ongoing process.	P06. Did you participate in at least two meetings with the team^a^ during which your goal(s) and goal attainment so far were discussed?
	S05. There are at least two meetings between P and the team^a^.	
Follow-up plan and continuity across levels of care	S09. P shall participate in preparing a specified written follow-up plan (aside from the epicrisis) for the follow-up process after the rehab period. This plan shall also include P's own efforts to maintain or improve function/health. S10. If there is a need for health care support after the rehab period, the relevant personnel are to be informed about the plan or participate in the development of the follow-up plan.	P09. Apart from regular epicrisis, was a written plan developed for the period after rehab, including what you were expected to work on yourself? (*if you have answered “yes” to this question, go to question P10. If you have answered “no” to this question, go to question P12*).
		P10. Did you participate in developing the plan?
		P11. As a part of this plan, were you consulted about whether you needed follow-up from external personnel^b^ after the rehab. period?
	S06. P is asked before meetings if he/she wants his/her next of kin to attend any of the meetings.	P07. Were you asked if you wanted your next of kin to attend any of the meetings?
	S07. P is asked before meetings if he/she wants some of the external professionals^b^ he/she will relate to after the rehab. to attend any of the meetings.	P08. Were you asked if you wanted professionals^b^ you will relate to after the rehab. period to attend any of the meetings?
Assessment, outcomes, and timepoint of evaluation	S08. The rehab unit uses reliable^c^ questionnaires and/or functional tests to assess physical, mental, and/or social conditions. P's goal/goal attainment is to be assessed… S11 …. at the beginning of the rehab period. S12. …at the end of the rehab period. S13. …3–6 months after the rehab period. P's function is to be registered… S14 … at the beginning of the rehab period. S15. … at the end of the rehab period. S16. …3–6 months after the rehab period. P's health-related quality of life is to be assessed… S17. … at the beginning of the rehab period. S18. … at the end of the rehab period. S19. …3–6 months after the rehab period.	P01. Were your health condition and life situation assessed during the first days of your rehab period? (Answer “no” if both aspects were not assessed) (*If you have answered “yes” to this question, go to question P02. If you have answered “no” to this question, go to question P03*).
		P02. Did the assessments include both a physical examination and questions about mental and social conditions, network, home situation, and—if relevant—your work situation?
		Outcome indicators (patient-reported): O12. As a result of the rehab period, have you achieved one or several goals that are important to you?
		O13. As a result of the rehab period, have you achieved an improvement in your physical, mental, and/or social functioning that is important to you?
		O14. As a result of the rehab period, do you think your quality of life has improved?

Sxx, structure indicator; Pxx, process indicator; Oxx, outcome indicator. P, the patient.

^a^ The interdisciplinary team or a professional representing the team.

^b^ Physiotherapist, general practitioner, a person from the labor welfare administration or the patient's workplace, if relevant for follow-up.

^c^ Reliable: quality-assured/validated questionnaires or tests.

All indicators are statements or questions requiring a dichotomous “yes” or “no” response, in which “yes” confirms the recommended indicator is fulfilled (“passed”). Summary pass rate for a complete questionnaire is calculated as the total number of indicators passed by each participant (institution or patient), divided by the total number of eligible items in the same questionnaire (20). Pass rate for a single indicator is calculated as the number of passed responses (“yes”) for that specific indicator, divided by the number of participants (institutions or patients) answering “yes” or “no” to the same indicator (20). Responses are reported as pass rates ranging from 0% to 100% (100 = the highest quality). The QI set has demonstrated feasibility, satisfactory face and content validity, and adequate responsiveness in both primary and secondary healthcare settings (20, 25).

We used an interrupted time series (ITS) design (26, 27) and hypothesized that systematic use of rehabilitation QIs and feedback on QI pass rates to providers would lead to better rehabilitation quality over time. The ITS approach was chosen for its suitability in clinical settings, as the quality of rehabilitation could be continuously monitored by collecting responses to the QI set from each patient throughout the study period. The feedback intervention was delivered simultaneously to all participating centres on the same date during the same meeting, making a clear differentiation of the pre- and post-intervention periods.

**Figure 1:**
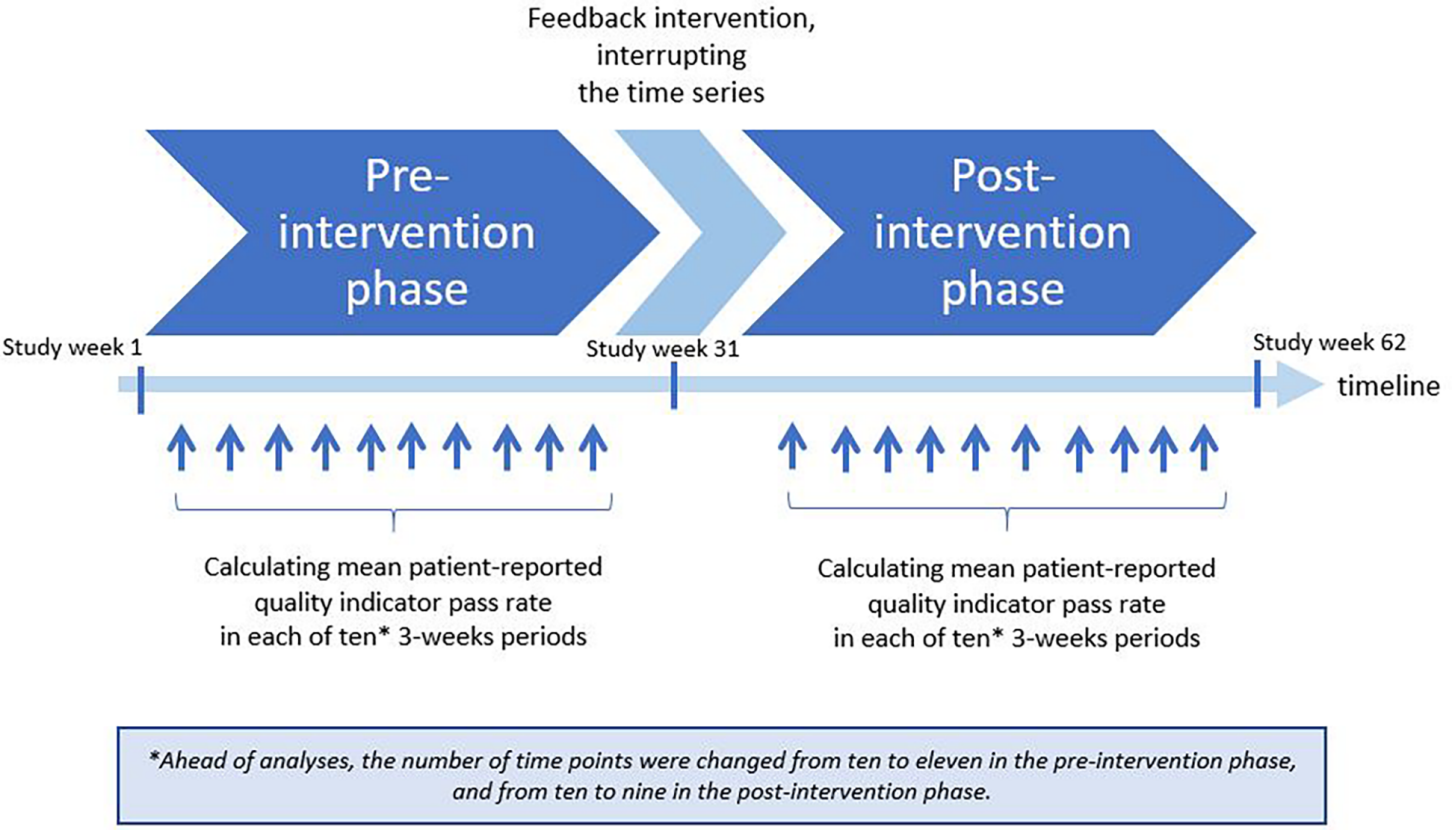
Timeline illustrating the planned interrupted time series design in the current study.

We defined three phases in the study: A pre-intervention phase (30 weeks), an intervention phase (1 week), and a post-intervention phase (30 weeks). The pre- and post-intervention phases were divided into consecutive 3-weeks intervals, as illustrated in Figure 1. Data on QIs collected from patients included in the first 30 weeks established the pre-intervention trend, while data from patients included in the last 30 weeks informed the post-intervention trend. In study week 31, the institutions received feedback on the QI pass rates. This provided an opportunity for the institutions to implement actions addressing areas in need for quality improvement, and thereby “interrupting” the pre-intervention pass rate trend. To assess the impact of the feedback intervention, we compared the post-intervention trend in QI pass rates with the pre-intervention trend, focusing on changes in both level and slope.

All participants provided written, informed consent before enrolment. Patient research partners and clinician representatives were involved in project development, design, and implementation of RehabNytte. The study was registered in ClinicalTrials.gov (NCT03764982), and recommended by the data protection officer at Diakonhjemmet Hospital (DS-00040, dated 17.10.2018). Approval from the Norwegian Regional Committee for Medical Research Ethics was not required (2018/1645/REK South-East A).

### Participants

Patient inclusion criteria were age ≥18 years, ability to read and understand questionnaires in Norwegian, access to a personal computer, tablet, or smartphone, and holder of a personal electronic credential for secure identification online. Exclusion criteria were severe cognitive impairment(s) or psychiatric disease(s) influencing the patient's ability to perform repeated self-reported assessments in a digital data base. This was necessary, as data collection in the overall RehabNytte relied on digital responses to patient-reported outcome measures (PROMs) at multiple time points (23).

### Intervention

The audit and feedback intervention targeted the clinical team members and their managers, acknowledging both parties as keys to delivering high-quality rehabilitation practice. The feedback was delivered as a 1-day (3.5 h) session organized by the RehabNytte Research and Development Network, and took place in a social context already known by the participants from earlier meetings in the group. The project leader first presented recommendations for high-quality rehabilitation practice as reflected in the rehabilitation QI set, addressing structures, processes, and patients' rehabilitation outcomes. Thereafter, she presented examples on how pass rate results can be used to identify and target areas in need of quality improvement, followed by suggestions on how to carry out various actions to improve written procedures or clinical delivery of rehabilitation tasks and processes. The participants engaged in successive reflections, discussing potential issues arising from suboptimal pass rates, and shared ideas on how to optimize their structures and clinical processes to address areas in need of improvement. Subsequently, the overall study-specific audit results were presented in plenum, in terms of mean pass rates for summary scores of complete questionnaires and single indicators, respectively. Institution-specific audit results were then conveyed confidentially, with written and graphical reports distributed to each institution. Overall, the institutions were encouraged to autonomously utilize the audit results, resolving and implementing feasible actions aimed at enhancing areas of suboptimal quality and sustaining high-quality areas in the post-intervention phase.

### Data collection and measurements

Each participant responded to the process and outcome indicators 3 months after admission to the rehabilitation institution. This approach allowed us to capture their perspectives on rehabilitation quality during rehabilitation in secondary care and the initial stages of the follow-up period at home. It also provided continuous measurement points throughout the pre- and post-intervention phases. Accordingly, a manager or team leader at each institution responded to the structure indicators in study week 5 and study week 46–51, using the QI questionnaire for providers. In this way, we measured the occurrence of written procedures for daily use addressing each step in the rehabilitation process from admission to follow-up, before and after the feedback intervention.

The outcome in our study was change in rehabilitation quality from pre- to post-intervention phase, measured by tracking and comparing the pre-and post- pass rate trends of patient-reported process- and outcome indicators, and comparing provider-reported QI pass rates for structure indicators before and after the feedback intervention.

At admission, the RehabNytte data collection system included sociodemographic variables, such as age, sex, diagnosis, comorbidities, education level, paid work and social security benefits. At admission, discharge, and after 3, 6 and 12 months, the patients responded to PROMs addressing various health aspects. The following variables were used only for analyses in other RehabNytte studies: work ability (using the Work Ability Score from the Work Ability Index) (28–30), pain level (11-point numeric rating scale), distribution of pain, duration of pain (31, 32), change in health status (using the Global Rating of Change Scale) (33), symptom acceptability (using the Patient Acceptable Symptom Scale) (34–36), and health related quality of life (using the EQ-5D-5l questionnaire) (37, 38). The QI questionnaire for process and outcome indicators was included in the data collection system at 3 months, and used as outcome in the current study. Within each 3 weeks period (termed an assessment point), data from individual patients were aggregated into group-level data based on mean pass rate for the patient-reported QIs collected in that period.

The length of the pre-intervention phase was adjusted from 30 to 33 weeks due to final decisions on date for the feedback session, and the length of the post-intervention phase was reduced from 30 to 27 weeks due to actions implemented by the Norwegian Ministries to combat the coronavirus outbreak. Consequently, the final time series consisted of 11 pre- and 9 post-intervention assessment points, numbered 0–10 and 11–19, respectively.

**Figure 2:**
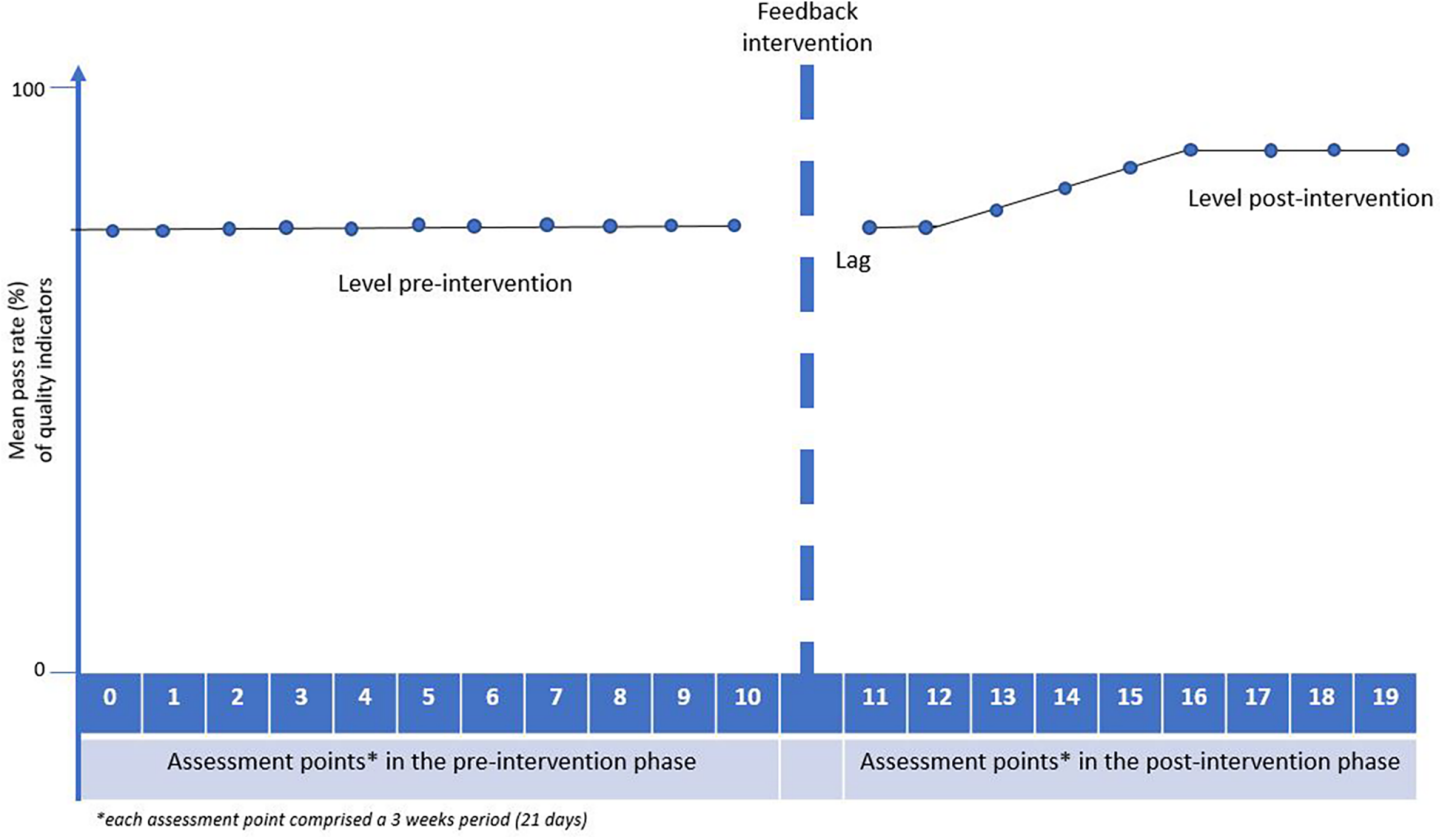
Before conducting the analyses, we expected a delayed but gradual improvement in quality following the feedback intervention.

We proposed an *a priori* impact model having a brief lag immediately after the feedback, followed by a temporary slope change, leading to a level change in rehabilitation quality later in the post-intervention phase (Figure 2). Hence, we expected the post-intervention changes in rehabilitation quality to follow gradually after a delay, as the institutions probably needed some time to respond to the feedback, and since improvements targeting some quality indicators may be easier to implement compared to more complex ones. This potential delay was acknowledged and included in analyses.

We used mixed effects models to address the hierarchical structure in our data set (having patients clustered in different rehabilitation centres, reporting at different assessment points) and to handle missing response data. Our main model included time as a continuous covariate, an indicator for the intervention period, and an interaction between these two. The random effects included centre specific intercepts and time slopes, and an autoregressive structure was assumed for the residuals. An additional model was used to simulate a potential delay in the intervention effect, using a linear spline at assessment points 12 and 16. Likelihood ratio tests between models with and without the post-intervention indicator variable were used to test a difference between the pre- and post-intervention periods.

The dependent variable in the analyses was the averaged patient-reported pass rate per centre at each assessment point. Analyses based on the pass rate for the complete patient-reported QI questionnaire was followed by separate analyses for the pass rate of the process- and outcome indicators, respectively. To adjust for individual patient attributes, similar mixed model analyses were used, however pass rates were not aggregated across individuals. These individual level analyses were adjusted for age, sex, body mass index, referral diagnosis, degree of comorbidity, smoking and/or snuff use, education level, paid employment, recipients of social security benefits, native language, civil status, caregiver, and annual gross income in the household.

We analysed data in STATA/IC version 16.0 and Microsoft Office Excel 2019. We considered *p*-values less than 0.05 as statistically significant.

## Results

### Provider-reported pass rates

All the participating centres were represented at the feedback intervention session.

In total, 29 managers or team leaders completed responses to the provider-reported questionnaires at the established pre- and post-intervention assessment points. This number was higher than the number of participating centres, as six multi-team centres implemented the audit- and feedback-intervention in more than one section within their institution. The observed summary pass rate (mean) across all centres and teams was 62.6% [95% confidence interval (CI) 54.8, 70.4] in the pre-intervention phase, compared to 69.9% (95% CI 62.2, 77.5) in the post-intervention phase.

**Figure 3:**
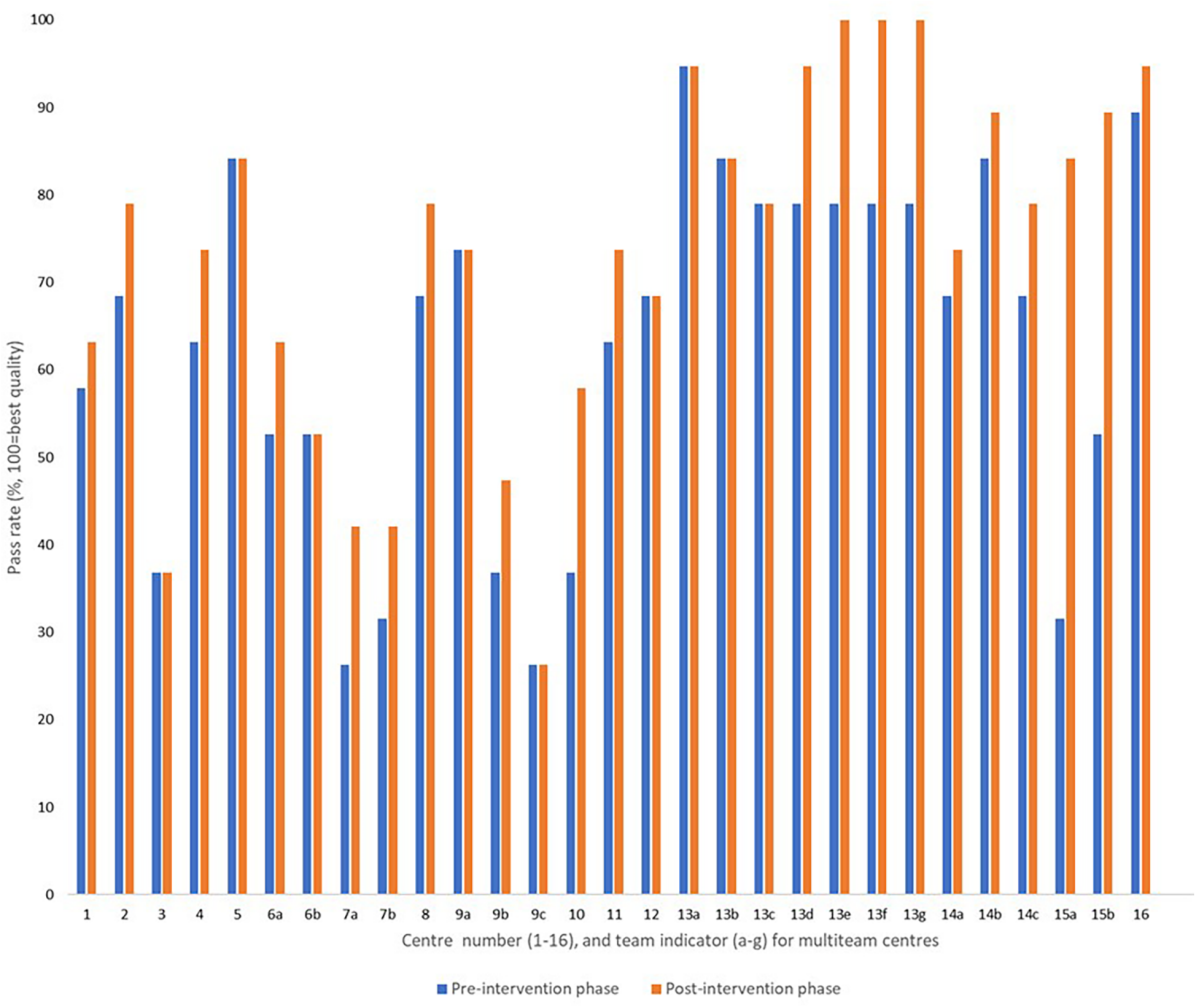
Provider-reported summary pass rates addressing the structure indicators before and after the feedback intervention, reported from 29 rehabilitation teams allocated to 16 institutions.

Nine teams reported no change in structure indicators (QIs S01–S19), 18 teams reported improvements in 1–4 structure indicators, and two teams reported improvements in 7 and 10 structure indicators, respectively (Figure 3). We observed a large variation (>68 percentage points) in structure quality among centres and teams in both phases (Figure 3).

### Patient-reported data

In total, 2,516 participants were recruited from 16 centres. Before analyses, we excluded participants included in the same week as the feedback intervention (*n* = 45), and participants included at the start of the pandemic (*n* = 56). The analyses were based on 2,415 participants distributed as 1,444 participants (59.8%) and 971 participants (40.2%) in the baseline pre- and post-intervention sample, respectively. The mean age of the total study sample was 52.1 years (±14 years), 68.9% were female, 52.3% had a rheumatic or musculoskeletal disease, and 56.3% were fully or partly employed. Baseline participant characteristics for the pre- and post-intervention samples are presented in Table 2.

**Table 2:** Baseline characteristics of participants (*n* = 2,415) and their allocation across institutions (*n* = 16).

Variable	Pre-intervention sample (*n* = 1,444)	Post-intervention sample (*n* = 971)
Age^a^, years, mean (SD)	52.2 (14.2)	51.9 (13.7)
Sex^a^, female, %	962 (66.6)	702 (72.3)
Referral diagnosis^b^, *n* (%)		
Rheumatic or musculoskeletal diseases	763 (52.8)	500 (51.5)
Cancer	75 (5.2)	49 (5.1)
Neurological disease	204 (14.1)	133 (13.7)
Lifestyle disease, overweight	170 (11.8)	100 (10.3)
Sensory impairment	55 (3.8)	55 (5.7)
Cardiovascular disease	56 (3.9)	37 (3.8)
Mental disease	29 (2.0)	36 (3.7)
Other disease	92 (6.4)	61 (6.3)
Rehabilitation institution^b^, *n* (%)		
Centre 1	55 (3.8)	54 (5.6)
Centre 2	152 (10.5)	118 (12.2)
Centre 3	31 (2.2)	38 (3.9)
Centre 4	106 (7.3)	52 (5.4)
Centre 5	66 (4.6)	51 (5.3)
Centre 6	209 (14.5)	157 (16.2)
Centre 7	131 (9.1)	78 (8.0)
Centre 8	32 (2.2)	5 (0.5)
Centre 9	28 (1.9)	13 (1.3)
Centre 10	93 (6.4)	72 (7.4)
Centre 11	26 (1.8)	20 (2.1)
Centre 12	39 (2.7)	18 (1.9)
Centre 13	105 (7.3)	67 (6.9)
Centre 14	188 (13.0)	99 (10.2)
Centre 15	64 (4.4)	44 (4.5)
Centre 16	119 (8.2)	85 (8.8)
Patient-reported data		
Comorbidities, *n*, median (min, max)	2 (0,10)	2 (0,9)
Body mass index, kg/m^2^, mean (SD)	30.2 (7.3)	29.7 (7.0)
Smoking and/or snuff use, *n* (%)	409 (28.3)	245 (25.2)
Education >12 years, *n* (%)	574 (39.8)	448 (46.1)
Paid work (currently, full or part time), *n* (%)	788 (54.6)	571 (58.8)
Recipients of social security benefits, *n* (%)	1,166 (80.8)	766 (78.9)
Language (native tongue), *n* (%)		
Norwegian, Swedish, or Danish	1,362 (94.3)	904 (93.1)
Other languages	79 (5.5)	64 (6.6)
Civil status, *n* (%)		
Married/cohabitant	780 (54.0)	575 (59.2)
Single	662 (45.8)	393 (40.5)
Caregiver for child(ren)/others in or outside home, *n* (%)	639 (44.3)	497 (51.2)
Annual gross income in the household >600,000 NKr, *n* (%)	636 (44.0)	481 (49.5)

SD, standard deviation.

^a^ Data collected from the national identification number.

^b^ Clinician-reporteddata.

The total baseline number of participants per assessment point ranged between 57 and 231 during the pre-intervention phase, and between 39 and 142 during the post-intervention phase (Additional file 1, Table A). Among the baseline participants, a total of 1,823/2,415 participants (75.4%) logged into the 3-month measurement time point. Of those, 1,777/1,823 participants (97.5%) answered the QI questionnaire, distributed as 1,078/1,104 (97.6%) participants in the pre-intervention sample, and 699/719 (97.2%) in the post-intervention sample. Among all institutions, the number of missing responses to the QI questionnaire was 2.5%, ranging from 0% to 6.7% (Additional file 1, Table B).

### Patient-reported pass rates and ITS analyses

At the individual participant level, the pre-intervention process pass rates ranged from 18.7% to 100%, compared to the range from 37.5% to 90.9% in the post-intervention phase. Outcome pass-rates ranged from 0% to 100% in both phases.

**Figure 4:**
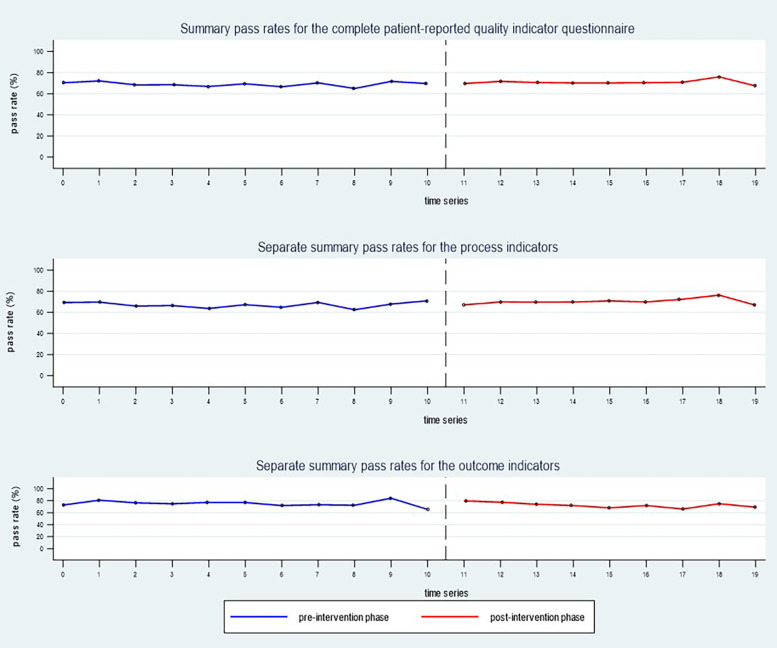
The observed summary pass rates (mean) for the whole study sample (*n* = 16 rehabilitation centres) during the pre-intervention phase (blue line) compared to the post-intervention phase (red line).

The observed summary pass rate (mean) for the complete patient-reported QI questionnaire was 68.8% in the total pre-intervention sample, compared to 70.7% in the total post-intervention sample. Separate analyses of the process indicators (P01–P11) revealed an observed pre-to-post-increase in pass rate (mean) from 66.7% to 70.4%. For the outcome indicators (O12–O14), the observed pass rate (mean) decreased from 75.2% in the pre-intervention phase to 72.4% post intervention (Figure 4). Estimated values for the complete study population are presented in Table 3, showing a small, statistically significant increase in summary pass rates for the process indicators. Unadjusted pass rates for each participating centre are presented in Additional file 2, showing no statistically significant differences between the pre- and post-intervention phases.

**Table 3:** Patient-reported pass rate changes from pre- to post-intervention phase, addressing process and/or outcome quality indicators.

Characteristic	Pre-intervention phase pass rate (%)	Post-intervention phase pass rate (%)	*p*-value*
	Mean [95% CI]	Mean [95% CI]	
Complete questionnaire			
Process and outcome indicators	68.8 [66.8, 70.7]	70.7 [69.0, 72.3]	0.17
Separate parts			
Process indicators	66.7 [64.5, 68.9]	70.4 [68.4, 72.4]	0.02
Outcome indicators	75.2 [72.1, 78.3]	72.4 [68.2, 76.6]	0.28

CI, confidence interval; *p*-value: significant at *p* < 0.05.

* Independent samples *t*-test.

In the mixed models analyses, we found that the trend in slope (time variable changes) was stable and almost unchanged, while the trend in intercept (pass rate changes) reflected a small level-change from pre- to post-intervention phase. However, the 95% CIs included the null value, and the adjusted mean differences between the pre- and post-intervention phases were not statistically significant (Table 4).

**Table 4:** Adjusted mean differences in patient-reported pass rate and time variable changes between the pre- and post-intervention phase, addressing process and/or outcome quality indicators.

Characteristic	Adjusted mean difference^a^ (percentage points), subtracting post—pre pass rates (%)	
	Mean [95% CI]	*p*-value
Complete questionnaire		
Pass rate change, process and outcome indicators	4.2 [−6.9, 15.3]	0.46
Time variable change, process and outcome indicators	−0.6 [−1.5, 0.3]	0.16
Separate parts		
Pass rate change, process indicators	6.4 [−5.2, 18.0]	0.28
Time variable change, process indicators	−0.9 [−1.8, 0.0]	0.06
Pass rate change, outcome indicators	−8.3 [−30.1, 13.5]	0.45
Time variable change, outcome indicators	0.4 [−1.3, 2.1]	0.66

CI, confidence interval; *p*-value: significant at *p* < 0.05.

^a^ Estimated with linear mixed models with linear time effect, random intercepts and random slope on time.

Results from the fully adjusted individual-level model showed similar small, non-significant results. The change in pass rates (*Δ* = post-pre) for the complete questionnaire had a mean difference of 2.6 percentage points (95% CI −2.7, 8.1; *p* = 0.34). For process indicators alone, the mean difference was 2.1 percentage points (95% CI −4.2, 8.3; *p* = 0.51), while outcome indicators showed a mean difference of −4.3 percentage points (95% CI −6.1, 15.7; *p* = 0.42).

The first likelihood-ratio test indicated that a random-intercept model, including centre specific intercepts and time slopes, showed a statistically significant improvement over a fixed-effects-only model. However, a more complex intercept-and-slope model did not show a statistically significant improvement compared to the intercept-only model. The next likelihood-ratio test indicated that the model with linear-spline trend, simulating a potential delay in the intervention effect, did not give any better fit to the data. The final likelihood-ratio test including the option for autoregressive errors in the more complex model, gave statistically non-significant results. Hence, the pass rates' intercepts varied from place to place, but the time gradient did not work differently in different institutions. In the complete multicentre study population, the audit and feedback intervention did not lead to any increase in rehabilitation quality over time.

### Single indicators

**Figure 5:**
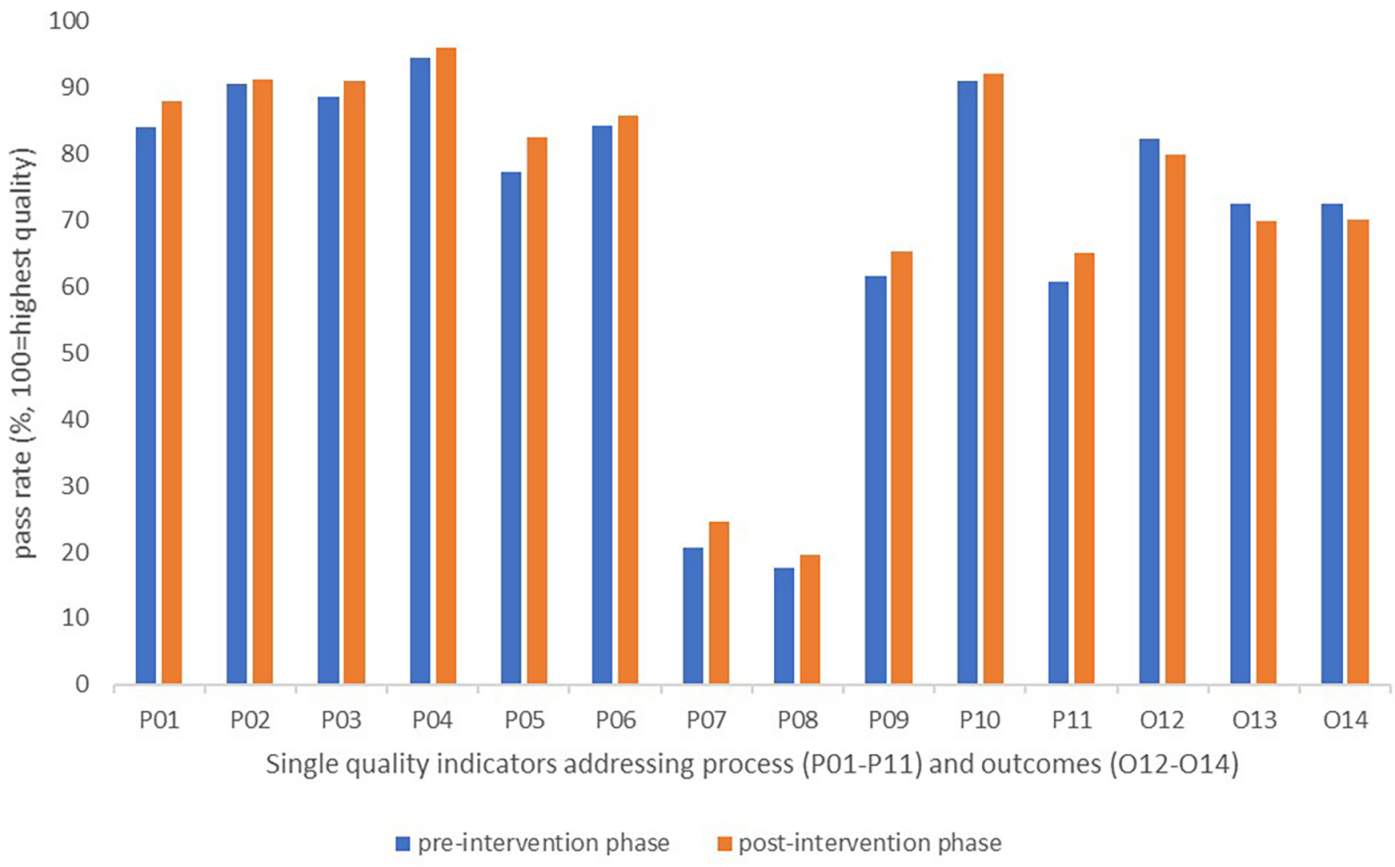
Changes in pass rates for single quality indicators reported by patients during the pre- and post-intervention phases.

At the level of single indicators, the observed pass rates increased from pre- to post-intervention phase for each of the process indicators (P01–P11), by 0.7–5.2 percentage points. For the single outcome indicators (O12–O14), we observed a reduction in pass rates by 2.3–2.8 percentage points (Figure 5).

Although small improvements occurred for the process dimension, the audit results after feedback remained lowest for process indicators targeting involvement of next of kin and/or external services in meetings with the rehabilitation team (P07 and P08). A persistent potential for improvement was also observed after feedback for the indicators targeting the use of a written plan for follow-up, and the involvement of external services in development of such plans (P09 and P11) (Figure 5).

After feedback, we also observed a persistent need for improvements targeting written procedures, because the pass rates remained low (<45%) for structure indicators targeting involvement of next of kin and/or external services (S06 and S07), and evaluation of progress after 3–6 months on goal attainment, function, and health-related quality of life (S13, S16, S19).

## Discussion

In this multicentre study, the audit and feedback intervention did not increase the quality of rehabilitation services, as measured by changes in quality indicator pass rates encompassing health service structures, processes and patient outcomes. The mixed model analyses revealed that the mean changes in patient-reported QIs between the pre- and post-intervention phase were small and statistically non-significant. The expected impact model, illustrating a gradually higher quality of rehabilitation following a brief lag after the feedback to institution managers and clinical team members, was not confirmed.

We consider the data collection for the audit to be feasible, with all institutions (*n* = 16) providing required structure indicator data pre- and post the feedback intervention, and over 97% of the participants responding to the process and outcome indicator questionnaire. Consequently, the study gave managers and team members a structured opportunity to critically reflect on their clinical rehabilitation practice, as experienced by their patients, and also to assess whether the intended delivery was clearly outlined in institution-specific written procedures for daily use. The importance of such opportunities to evaluate the quality of underlying structures and current practice performances have also been valued by health professionals in other audit and feedback studies (39, 40). However, gaining insight into their own service quality and benchmarking against relevant others does not guarantee that managers or clinical team members automatically will translate this into targeted quality improvement actions (40, 41). While attention to a discrepancy between actual and recommended performance constitutes the core mechanism of audit and feedback interventions, the subsequent response actions at each centre must align with local context and resources available for quality improvement (15, 39, 41).

The managers and clinical team members in our study were encouraged to autonomously utilize the audit results and implement actions applicable and feasible at each centre. Autonomous feedback response has also been used in previous audit and feedback interventions, without detailed instructions to the recipients of the feedback about how to change their practice (12, 15, 39). Some institutions likely have an established system for continuous learning and quality improvement, enabling them to autonomously develop their feedback responses to target the analysed pass rate results, and implement actionable improvements (42, 43). However, it takes time to build cultures for learning and improvement within the health system, and the field of rehabilitation is encouraged to pay more attention to the quality of care (42, 44). In a large, cluster-randomized trial from The Netherlands, an audit and feedback intervention yielded no improvement of multidisciplinary, clinical performance measured by quality indicators for cardiac rehabilitation (45). This aligns with our findings, although the Dutch audit and feedback intervention included additional components such as outreach visits, repeated feedback reports, and both measurement and adjustment of local goal setting and action plans for quality improvement (46). Presumably, the team members struggled to translate their intentions into concrete actions. Organizational barriers may also have contributed to the ineffectiveness, such as limited organizational readiness for change, insufficient time, competing priorities, and suboptimal local capacity, skills or knowledge on how to improve their service delivery (45, 47–49). In particular when institutions are granted autonomy in responding to feedback, they may need a longer time interval to plan and implement their improvement initiatives (39). This highlights the need for a deeper understanding of how to establish local quality improvement goals, and effectively accomplish the necessary actions to achieve them within a local rehabilitation setting (45, 47).

The lack of improvement observed in our study may also be attributed to the complexity of rehabilitation as an intervention. It has been suggested that audit and feedback tend to be more effective for simpler changes in healthcare performance compared to more complex interventions, such as rehabilitation (12, 15). However, for complex interventions in particular, institutions should identify the distinct interacting components of the entire rehabilitation process, establish written procedures for implementing them within their specific context, and evaluate how health professionals practice the intervention in that clinical setting (50, 51). The QI set used as audit in our study is designed to evaluate the complex rehabilitation intervention through measurable and distinct elements encompassing written procedures, clinical processes, and patient outcomes (20). Indicators related to structures and processes are often more within the control of managers and team members, providing greater opportunities for quality improvement compared to those related to patient outcomes (52–54). Despite this, we observed only modest improvements in structure and process pass rates, and the estimated values of these changes were not statistically significant. Although we did not examine reasons for improvement or lack thereof, it is possible that our feedback recipients perceived the total number of structure and process indicators as overwhelming. Such perceptions may have diminished their motivation to focus on enhancing the quality in fewer, prioritized areas of rehabilitation delivery. Other researchers and behaviour change theories propose that audit and feedback interventions are more likely to be effective when recipients are highly motivated and can identify an appropriate number of achievable targets (12, 15, 52).

Receiving institution-specific audit results, accompanied by benchmark comparisons, was anticipated to improve the institutions' ability to take actionable steps, particularly in cases of low pass rates. Contrary to this assumption, we found no significant improvement in the indicators with the greatest potential for improvement. Although rehabilitation is recognized as a longitudinal trajectory spanning across levels of care, the post-intervention pass rates remained low for indicators related to key aspects of follow-up. These include the development of written plans for follow-up, involvement of next of kin and external services, and the evaluation of progress in patients' goal attainment, functional outcomes, and health-related quality of life during the follow-up period. Our results align with previous research indicating that feedback recipients do not always target indicators with obvious room for improvement (47, 55). In these studies, health professionals overlooked the potential for improvement, either because they considered the pass rates sufficiently high, or they did not regard the indicator as an essential part of rehabilitation quality. Other reported reasons include the perceived infeasibility of improving the indicator, or the lack of organizational support to address the area reflected by the indicator (47, 55). Organizational support is likely to be particularly critical in the area of follow-up, where immediate improvements may be difficult and beyond the control of clinical team members. Instead, progress in this area often depends on decisions made by managers across levels of care.

Participating teams in our study reported a range of outcomes, from minimal or no improvement to more substantial gains in pass rates. This variation may partly be explained by the types of benchmarking used. Previous research suggests that benchmarks, such as the QI set's ideal pass rate of 100% and overall institutional average, may have been too high for some teams with low audit results, particularly those with limited prior experience in quality improvement efforts (56). Conversely, teams with high audit results might have dismissed the feedback if their performance was already at or above the average. For such teams, benchmarking solely against the ideal pass rate of 100% or the top 10% might be more appropriate (56). A review on audit design recommends incorporating tailored feedback messages and setting performance targets at different levels to better address the specific needs of individual institutions in future audit and feedback interventions (56).

Strengths of this study include its robust quasi-experimental ITS design, which is regarded as a valuable tool for quality improvement initiatives (27). The potential of a gradual implementation phase was included in the impact model, as well as in the mixed model analyses (26). The study utilized 20 assessment points, each spanning a 3 weeks interval, deemed sufficient for capturing potential improvements within the predefined data collection period of the RehabNytte project. The study's power may have been limited due to the adjustment in number of assessment points, with 11 conducted before and 9 after the intervention, deviating from the originally planned equal distribution. Additionally, the power may have been further constrained by a final sample size smaller than the planned recruitment target of 4,000 participants, which was intended to ensure a sufficient number of participants per assessment point. Seasonality, indicated by the uneven distribution of spring and summer months before and after the feedback session, along with concurrent events such as the interruption by the COVID-19 pandemic, may have introduced bias into the results (26). Because patient-reported responses to the quality indicators were not assessed at discharge, there is a potential for recall bias. However, the first at-home measurement time point (T3) was preferred, as the indicators addressed not only the inpatient stay but also the use of rehabilitation plans and the involvement of external services during the follow-up period. Finally, although six centres provided data from multiple teams, the patient data were collected only at the centre level, making it challenging to attribute patient data to specific teams. A more thorough planning of this aspect could have improved the study.

Although the QI set is not yet widely adopted, it strengthens the study that this audit tool has been systematically designed for multidisciplinary rehabilitation services, drawing on evidence from the literature and informed by expert consensus (20). We intentionally designed the feedback session to be straightforward and short, allowing participating institutions to integrate their feedback responses into their existing quality improvement routines. Our study did not examine the acceptability of the feedback reports or how the reports were utilized by institution managers and clinical team members, as the available time and funding limited the study's scope. However, incorporating a cyclic and iterative feedback process could have strengthened the intervention in this study (14).

## Conclusion

Using the ITS approach, we evaluated an audit and feedback intervention aimed at improving the quality of multidisciplinary rehabilitation services in a heterogeneous secondary healthcare setting in Norway. Despite continuous auditing of quality indicators and providing feedback to participating institutions, the intervention did not result in significant improvements in the structure, process, or outcome dimensions of service quality. Our study revealed variations in service quality across institutions, also during the post-intervention phase. We therefore encourage future research to focus on refining feedback mechanisms and quality improvement processes. In particular, we advocate for deeper exploration into the development of locally defined performance goals, behaviours, and actions to optimize the quality of rehabilitation services.

## Data Availability

The datasets presented in this article are not readily available because availability is restricted to parts with permission from the Norwegian Regional Committee of Medical Research Ethics. Requests to access the datasets should be directed to Anne-Lene Sand-Svartrud, anne-lene.svartrud@diakonsyk.no.
