# Supplementary material for: Impact of provider feedback on quality improvement in rehabilitation services: an interrupted time series analysis
Source: Front Rehabil Sci. 2025 Mar 6;6:1564346. doi: 10.3389/fresc.2025.1564346 (PMC11925202; doi:10.3389/fresc.2025.1564346)
Supplement: Supplementary file 1 [file Datasheet1.pdf]

Additional file 1

Table A The baseline number of participants per institution per assessment period during the pre-(n=1444) and post-intervention phase (n=971).

	3-week periods (number 0-10) comprising the pre-intervention phase											
Institution n	period 0	period 1	period 2	period 3	period 4	period 5	period 6	period 7	period 8	period 9	period 10	Total
1	17	4	11	8	1	8	0	0	0	0	6	55
2	32	23	13	16	13	16	7	9	4	5	14	152
3	6	9	8	8	0	0	0	0	0	0	0	31
4	15	14	15	12	9	6	4	12	10	7	2	106
5	10	5	3	10	6	6	10	7	1	0	8	66
6	22	23	25	20	16	25	22	23	10	7	16	209
7	18	11	12	17	22	13	11	9	7	4	7	131
8	8	2	5	5	1	1	3	3	1	3	0	32
9	10	4	0	4	0	0	3	2	3	0	2	28
10	18	13	11	11	7	6	12	6	4	0	5	93
11	5	8	3	2	2	1	2	1	2	0	0	26
12	7	4	1	4	4	9	4	4	2	0	0	39
13	10	16	13	12	3	14	11	8	4	3	11	105
14	16	49	15	7	8	32	11	5	4	40	1	188
15	12	7	5	13	3	9	4	3	5	0	3	64
16	25	10	16	10	7	9	14	11	0	7	10	119
**Total**	231	202	156	159	102	155	118	103	57	76	85	**1444**

	3-week periods (number 11-19) comprising the post-intervention phase									
Institution n	period 11	period 12	period 13	period 14	period 15	period 16	period 17	period 18	period 19	Total
1	8	12	0	6	10	0	7	11	0	54
2	7	18	18	10	17	1	16	16	15	118
3	0	11	11	6	0	0	0	6	4	38
4	1	6	8	5	9	8	5	4	6	52
5	9	7	6	3	2	3	4	13	4	51
6	17	21	18	24	20	9	25	16	7	157
7	11	10	8	8	8	6	5	8	14	78
8	0	3	1	1	0	0	0	0	0	5
9	0	2	1	0	4	0	4	2	0	13
10	5	12	9	12	7	1	4	13	9	72
11	5	1	1	4	4	1	0	3	1	20
12	4	3	0	5	1	0	2	0	3	18
13	9	8	11	9	10	0	8	4	8	67
14	20	9	26	5	1	3	2	26	7	99
15	3	7	0	6	5	0	2	11	10	44
16	20	0	22	9	0	7	10	9	8	85
**Total**	119	130	140	113	98	39	94	142	96	**971**

Table B Among the participants who logged into the 3-month measurement time point after baseline (1823/2415 participants), a high number responded to the quality indicator questionnaire.

	The proportion of responders	The number of non-responders		The proportion of non-responders
**Institution number**	In total	Pre-intervention phase	Post-intervention phase	In total
1	83/85 (97.6%)	2	0	2/85 (2.4%)
2	179/182 (98.4%)	1	2	3/182 (1.6%)
3	54/54 (100%)	0	0	0 %
4	123/125 (98.4%)	1	1	2/125 (1.6%)
5	79/83 (95.2%)	2	2	4/83 (4.8%)
6	266/279 (95.3%)	7	6	13/279 (4.7%)
7	167/169 (98.8%)	1	1	2/169 (1.2%)
8	32/32 (100%)	0	0	0 %
9	34/34 (100%)	0	0	0 %
10	129/131 (98.5%)	1	1	2/131 (1.5%)
11	28/30 (93.3%)	1	1	2/30 (6.7%)
12	47/48 (97.9%)	1	0	1/48 (2.1%)
13	123/124 (99.2%)	0	1	1/124 (0.8%)
14	212/215 (98.6%)	3	0	3/215 (1.4%)
15	85/89 (95.5%)	4	0	4/89 (4.5%)
16	136/143 (95.1%)	2	5	7/143 (4.9%)
**Total**	**1777/1823 (97.5%)**	**26**	**20**	**46/1823 (2.5%)**
