# Supplementary material for: Impact of provider feedback on quality improvement in rehabilitation services: an interrupted time series analysis
Source: Front Rehabil Sci. 2025 Mar 6;6:1564346. doi: 10.3389/fresc.2025.1564346 (PMC11925202; doi:10.3389/fresc.2025.1564346)
Supplement: Supplementary file 2 [file Datasheet2.pdf]

Additional file 2

Table A Patient-reported pass rate changes from pre- to post-intervention phase, addressing process or outcome quality indicators.

Centre	Characteristic	Pre-intervention phase pass rate Mean [95% CI]	Post-intervention phase pass rate Mean [95% CI]	p-value[1]
Centre 1	Process indicators	71.9 [66.2, 77.6]	70.1 [67.8, 72.4]	0.51
	Outcome indicators	76.0 [59.2, 92.8]	77.4 [65.0, 89.9]	0.87
Centre 2	Process indicators	70.2 [65.1, 75.3]	76.3 [72.0, 80.5]	0.06
	Outcome indicators	69.3 [60.1, 78.5]	65.6 [49.7, 81.4]	0.63
Centre 3	Process indicators	55.3 [38.6, 71.9]	68.4 [56.8, 80.0]	0.09
	Outcome indicators	95.2 [80.1, 100.0]	89.2 [70.0, 100.0]	0.52
Centre 4	Process indicators	65.7 [60.3, 71.1]	66.9 [58.7, 75.0]	0.78
	Outcome indicators	85.3 [76.0, 94.6]	68.9 [47.2, 90.5]	0.11
Centre 5	Process indicators	81.9 [76.3, 87.6]	72.5 [63.6, 81.4]	0.05
	Outcome indicators	82.6 [71.6, 93.6]	72.2 [51.0, 93.5]	0.32
Centre 6	Process indicators	73.0 [70.0, 76.0]	70.0 [64.1, 75.7]	0.27
	Outcome indicators	73.8 [67.7, 79.9]	71.4 [63.3, 79.5]	0.59
Centre 7	Process indicators	54.7 [46.9, 62.4]	60.3 [53.4, 67.2]	0.24
	Outcome indicators	68.5 [57.1, 79.9]	72.6 [64.4, 80.7]	0.54
Centre 8	Process indicators	62.3 [50.5, 74.1]	71.2 [61.3, 81.1]	0.39
	Outcome indicators	77.5 [60.5, 94.5]	72.2 [0.0, 100.0]	0.76
Centre 9	Process indicators	67.8 [59.5, 76.2]	74.7 [67.8, 81.6]	0.16
	Outcome indicators	59.5 [27.9, 91.1]	73.3 [38.7, 100.0]	0.48
Centre 10	Process indicators	59.2 [51.8, 66.5]	68.7 [60.8, 76.6]	0.06
	Outcome indicators	77.8 [65.7, 90.0]	67.5 [44.8, 90.2]	0.36
Centre 11	Process indicators	76.4 [62.5, 90.4]	67.5 [35.0, 100.0]	0.43
	Outcome indicators	70.5 [47.4, 93.7]	77.8 [0.0, 100.0]	0.73
Centre 12	Process indicators	44.2 [31.9, 56.6]	62.4 [42.3, 82.5]	0.07
	Outcome indicators	70.8 [45.9, 95.8]	92.1 [79.3, 100.0]	0.13
Centre 13	Process indicators	78.9 [74.4, 83.4]	83.2 [79.5, 87.0]	0.13
	Outcome indicators	66.3 [54.4, 78.3]	55.7 [43.1, 68.3]	0.19
Centre 14	Process indicators	62.2 [52.9, 71.5]	68.8 [62.0, 75.7]	0.23
	Outcome indicators	90.5 [83.8, 97.2]	92.1 [83.5, 100.0]	0.74
Centre 15	Process indicators	68.2 [58.9, 77.5]	74.4 [69.4, 79.4]	0.25
	Outcome indicators	74.3 [62.9, 85.8]	50.9 [28.0, 73.7]	0.31
Centre 16	Process indicators	67.5 [62.2, 72.9]	70.4 [61.6, 79.1]	0.50
	Outcome indicators	70.6 [60.4, 80.8]	72.4 [59.7, 85.2]	0.80

[1]*Independent samples t-test. CI: confidence interval; p-value: significant at* $p < 0.05$.
